# Cdc48 and Cofactors Npl4-Ufd1 Are Important for G1 Progression during Heat Stress by Maintaining Cell Wall Integrity in *Saccharomyces cerevisiae*

**DOI:** 10.1371/journal.pone.0018988

**Published:** 2011-04-19

**Authors:** Meng-Ti Hsieh, Rey-Huei Chen

**Affiliations:** 1 Molecular Cell Biology, Taiwan International Graduate Program, Institute of Molecular Biology, Academia Sinica, and Graduate Institute of Life Sciences, National Defense Medical Center, Taipei, Taiwan; 2 Institute of Molecular Biology, Academia Sinica, Taipei, Taiwan; Texas A&M University, United States of America

## Abstract

The ubiquitin-selective chaperone Cdc48, a member of the AAA (ATPase Associated with various cellular Activities) ATPase superfamily, is involved in many processes, including endoplasmic reticulum-associated degradation (ERAD), ubiquitin- and proteasome-mediated protein degradation, and mitosis. Although Cdc48 was originally isolated as a cell cycle mutant in the budding yeast *Saccharomyces cerevisiae*, its cell cycle functions have not been well appreciated. We found that temperature-sensitive *cdc48-3* mutant is largely arrested at mitosis at 37°C, whereas the mutant is also delayed in G1 progression at 38.5°C. Reporter assays show that the promoter activity of G1 cyclin *CLN1*, but not *CLN2*, is reduced in *cdc48-3* at 38.5°C. The cofactor *npl4-1* and *ufd1-2* mutants also exhibit G1 delay and reduced *CLN1* promoter activity at 38.5°C, suggesting that Npl4-Ufd1 complex mediates the function of Cdc48 at G1. The G1 delay of *cdc48-3* at 38.5°C is a consequence of cell wall defect that over-activates Mpk1, a MAPK family member important for cell wall integrity in response to stress conditions including heat shock. *cdc48-3* is hypersensitive to cell wall perturbing agents and is synthetic-sick with mutations in the cell wall integrity signaling pathway. Our results suggest that the cell wall defect in *cdc48-3* is exacerbated by heat shock, which sustains Mpk1 activity to block G1 progression. Thus, Cdc48-Npl4-Ufd1 is important for the maintenance of cell wall integrity in order for normal cell growth and division.

## Introduction

Budding yeast Cdc48 and its metazoan homolog p97, also named as valosin-containing protein (VCP), are abundant and evolutionarily conserved proteins. Cdc48/p97 belongs to the AAA ATPase superfamily and is involved in many aspects of cellular activities, including homotypic membrane fusion of organelles [1], ERAD [2], ubiquitin/proteasome-mediated protein degradation [3], and cell cycle control [4].

The diverse functions of Cdc48/p97 are mediated by specific cofactors. The binary complex Npl4-Ufd1 is associated with ER membrane and required for degradation of ER proteins [5]. Npl4 contains NZF domain that binds polyubiquitin chain [6]. The N-terminal domain of Ufd1 also has a higher affinity toward polyubiquitin than monoubiquitin [7]. Cdc48 coupled with Npl4-Ufd1 functions in retrograde translocation of proteins from ER for degradation (ERAD) [8]. Cdc48/p97 also binds a family of proteins containing a ubiquitin-related (UBX) domain that is structurally similar to ubiquitin [9]. Ubx1, also known as Shp1 (Suppressor of high copy protein phosphatase 1) [10], Ubx2, Ubx4, Ubx6, and Ubx7 serve as cofactors for Cdc48 in ubiquitin-dependent protein degradation [11]. Cdc48-Shp1 is also important for chromosome bi-orientation [12]. On the other hand, the mammalian homolog of Shp1, p47, is involved in membrane fusion [13].

Budding yeast Cdc48 was originally isolated as a cell cycle mutant that arrested in mitosis at the restrictive temperature [4]. Cdc48/p97 appears to have multiple functions in the cell cycle. In budding yeast, Cdc48 is required for passing Start, the cell cycle commitment point in G1, by degrading the G1-cyclin-dependent kinase inhibitor Far1 [14]. In fission yeast, Cdc48 is required for the metaphase-to-anaphase transition by stabilizing Separase [15], the enzyme that cleaves cohesin components to separate sister chromatids. We have previously demonstrated that budding yeast Cdc48 and its cofactor Shp1 promote chromosome bi-orientation by balancing Aurora B activity [12]. In addition, Cdc48/p97 together with Npl4-Ufd1 has been shown to participate in spindle disassembly during mitotic exit [16], although the result is controversial [17]. p97 is also important for the formation of a closed nuclear envelope and nuclear expansion following nuclear envelope formation [18]. Cdc48/p97 itself is regulated in the cell cycle. The protein is primarily associated with membranes of organelles such as the ER and the Golgi [1]. In G1 phase, a fraction of Cdc48 enters the nucleus in a phosphorylation-dependent manner [19]. The change of Cdc48 localization during the cell cycle likely reflects its multiple functions.

Cell cycle progression is mainly governed by cyclin-dependent kinases (CDK). Coupled with G1 or mitotic cyclins, the CDK activity drives G1/S transition or mitotic entry, respectively. Budding yeast has three G1 cyclins encoded by *CLN1*, *CLN2*, and *CLN3* [20]. These G1 cyclins share redundant functions, as cells can live on just one of the cyclins [21]. The expression of these genes is induced as cells traverse G1. The mRNA and protein of *CLN3* constantly exist during the cell cycle and are modestly induced at late G1 [22]. On the other hand, Cln1 and Cln2 are present at low levels at early G1 and transiently induced before Start [20], [22]. The induction of *CLN1* and *CLN2* is mediated through the SCB and MCB sequences in their promoters that bind transcription factors Swi4/Swi6 (SBF) and Swi4/Mbp1 (MBF) complexes, respectively [23]. Activation of both SBF and MBF is dependent on the kinase activity of Cln3-Cdc28 [23]. Cln3-Cdc28 also inactivates Whi5, the transcription suppressor that inactivates SBF specifically by direct binding until G1 [24], [25]. Thus, Cln proteins promote their own expression through a positive feedback loop [26], [27], [28].

The Start commitment point in G1 phase of budding yeast is controlled by nutrient availability, cell size, and the presence of mating pheromone [29]. In addition, heat shock transiently inhibits Start [30]. Heat shock generates misfolded or aggregated proteins that trigger heat shock response pathway featuring the induced synthesis of a set of evolutionarily conserved heat shock proteins [31]. Many of the heat shock proteins are chaperones that help protein folding [31]. The induction of heat shock proteins are primarily mediated by heat shock factor (HSF) and transcription factors Msn2 and Msn4 that bind to heat shock elements (HSE) and stress response element (STRE), respectively, in the promoter of many heat-inducible genes [32]. In addition, heat shock activates the MAP kinase homolog Mpk1 that maintains cell wall integrity and prevents cell lysis when cells are grown at elevated temperature [33]. Mpk1 is downstream of Pkc1 that regulates a protein kinase cascade in which Bck1 (MEK kinase) activates Mkk1 and Mkk2 (MAP kinase-kinase) that in turn activate Mpk1 [34]. This cell wall integrity pathway senses the cell surface defects through cell surface proteins Wsc1 [35] and Mid2 [36]. Mpk1 phosphorylates and activates transcription factor Rlm1 that regulates the expression of many genes involved in the cell wall biogenesis [37]. Mpk1 is also important for the heat shock-induced transcription through the HSE and STRE elements [33]. In addition, the SBF transcription factor is a target of Mpk1 [38], providing a link between heat stress and cell cycle control.

In this study, we examine the cell cycle function of Cdc48 and show that Cdc48 and the cofactor Npl4-Ufd1 complex are important for maintaining the cell wall integrity during heat stress to allow G1 progression.

## Results

### G1 delay of *cdc48-3* at high temperature

**Figure 1:**
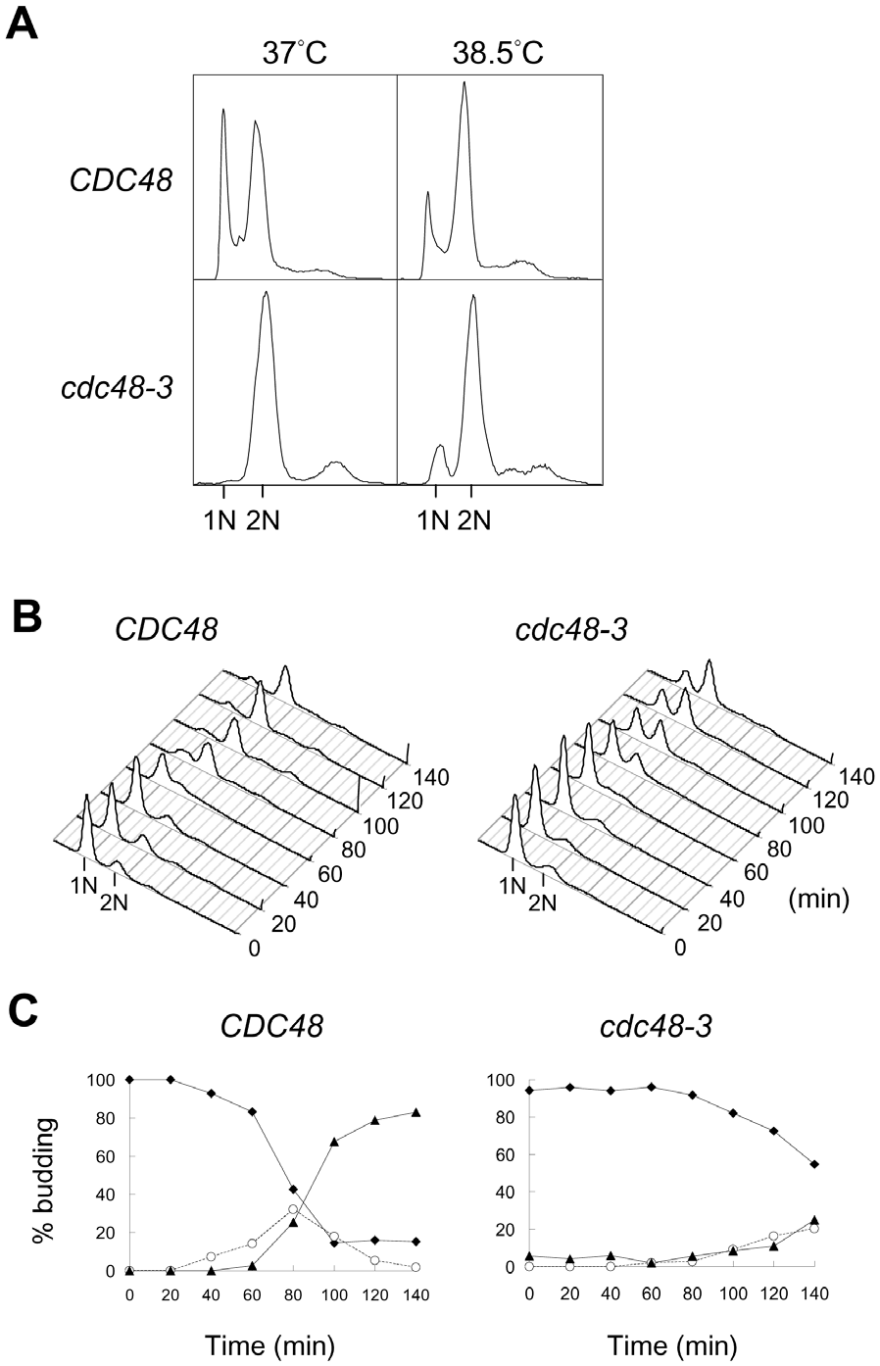
*cdc48-3* is delayed in G1 progression at high temperature. (A) *CDC48* and *cdc48-3* cells were grown at 37°C or 38.5°C for 4 hr and then analyzed by FACS. (B) *CDC48* and *cdc48-3* cells were first arrested at G1 with α-factor. The cells were shifted to 38.5°C during the last 30 min of the arrest, and then released into the cell cycle at 38.5°C. Samples were taken at the indicated times after the release for FACS analysis. (C) Cells were grown as described in (B) and their budding index at the indicated times during the cell cycle entry were determined. Filled diamond, no bud; open circle, small bud; filled triangle, medium/large bud.

In order to understand the cell cycle function of Cdc48, we have examined the phenotypes of the temperature-sensitive *cdc48-3* mutant. The mutant was largely arrested at mitosis with 2N DNA content at 37°C as determined by fluorescence-activated cell sorter (FACS) analysis (Fig. 1A). Interestingly, a small fraction of cells contained 1N DNA at 38.5°C, indicating that G1 progression was perturbed. To analyze the cell cycle progression, we first arrested the cells at G1 with α-factor and then released the arrest at 38.5°C. FACS analysis showed that most of the wild-type cells completed DNA replication by 100 min after the release (Fig. 1B). However, less than 50% *cdc48-3* cells completed DNA replication by 100 min and some cells still contained 1N DNA even at 140 min (Fig. 1B). Examination of the cell morphology showed that more than 50% of wild-type cells have budded by 80 min after release from α-factor arrest and more than 80% of the cells have become large-budded after 140 min (Fig. 1C). However, only ∼50% of *cdc48-3* cells have budded with only 20% large-budded cells at 140 min (Fig. 1C). These results show that *cdc48-3* mutant was delayed at G1/S transition at 38.5°C.

### Reduced expression of G1 cyclin

**Figure 2:**
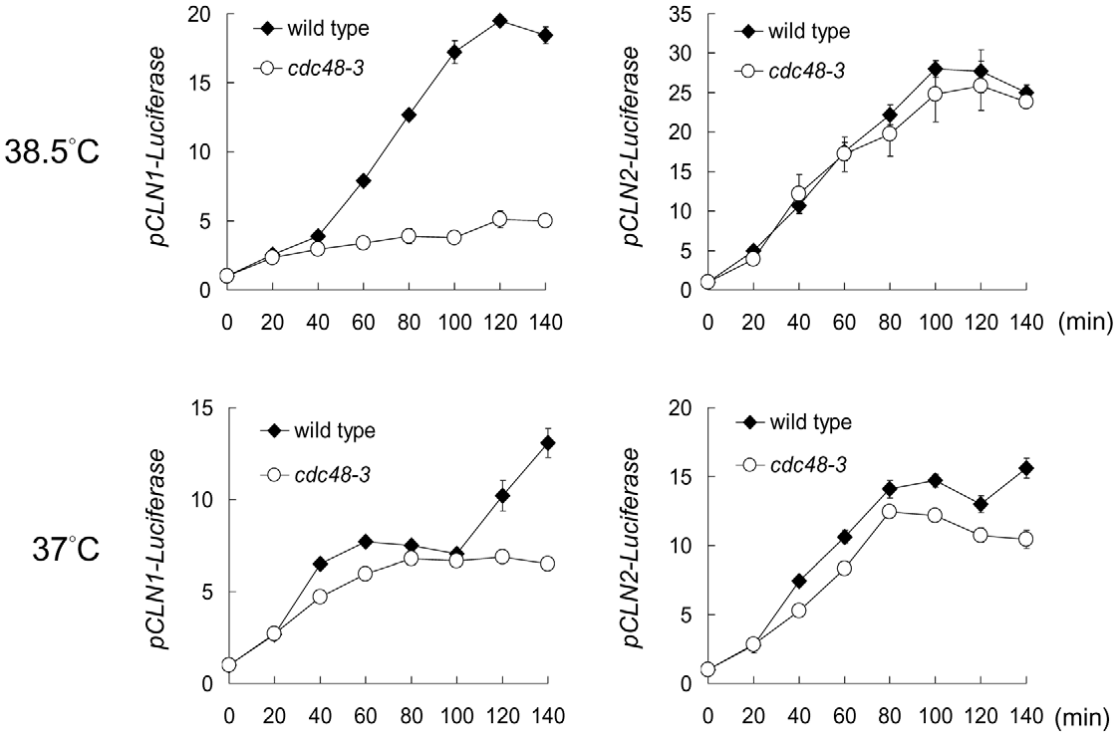
*CLN1* promoter activity is reduced in *cdc48-3* at high temperature. Wild-type and *cdc48-3* cells carry *Renilla reniformis* and *Pyrophorus plagiophthalamus* luciferases under the control of *CLN1* and *CLN2* promoters, respectively. Cells were first arrested at G1 with α-factor, and then released into the cell cycle at 38.5°C or 37°C. Luciferase activities were measured in triplicates at the indicated times after the release. The activities were normalized to that at time 0. The plot shows the average activities in fold increase and the standard deviation.

G1 progression is controlled by the accumulation of G1 cyclins encoded by *CLN1*, *CLN2*, and *CLN3*. Because *CLN1* and *CLN2* are transiently induced before G1/S transition, we measured the promoter activities of *CLN1* and *CLN2* during a synchronous cell cycle by reporter assays. Figure 2 shows that the luciferase activity driven by the *CLN1* promoter increased about 20 folds at 120 min after release from G1 arrest in the wild-type cells at 38.5°C, whereas the activity increased only 5 folds in *cdc48-3*. *CLN1* promoter activity was slightly lower in *cdc48-3* than that in the wild-type cells at 37°C during 100 min after release from G1 arrest (Fig. 2). The rise of the activity after 100 min in the wild-type cells likely reflected the second cell cycle. *cdc48-3* cells did not show further increase of *CLN1* promoter activity after 100 min, because the mutant was arrested at mitosis at 37°C [12]. Unlike *CLN1* promoter, *CLN2* promoter-driven luciferase activities were comparable in the wild-type and *cdc48-3* cells at both 38.5°C and 37°C (Fig. 2). This result suggests that *CLN1*, but not *CLN2*, promoter activity was affected in *cdc48-3* at 38.5°C.

**Figure 3:**
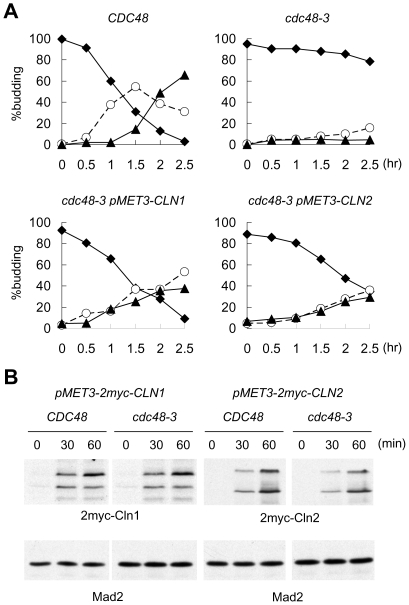
Ectopic expression of either Cln1 or Cln2 promotes G1 progression in *cdc48-3*. (A) *CDC48*, *cdc48-3*, and *cdc48-3* expressing 2myc-Cln1 or 2myc-Cln2 from *MET3* promoter were first arrested at G1 with α-factor in synthetic medium containing methionine. The cells were shifted to 38.5°C during the last 30 min of arrest, and then released from the arrest in methionine-free medium to induce the expression of 2myc-Cln1 or 2myc-Cln2. Budding index was determined at the indicated times after the release. Filled diamond, no bud; open circle, small bud; filled triangle, medium/large bud. (B) Cells from the above experiment were taken at the indicated times for Western blots with anti-myc antibody to detect 2myc-Cln1 and 2myc-Cln2. Mad2 blot serves as a loading control.

The reduced *CLN1* promoter activity in *cdc48-3* at 38.5°C suggests that the G1 delay may result from reduced levels of G1 cyclins. To test this possibility, we expressed *CLN1* or *CLN2* through the *MET3* promoter in *cdc48-3*. The cells were released from G1 arrest in methionine-free medium to induce *CLN1* or *CLN2* expression. Without additional *CLN1* or *CLN2*, *cdc48-3* cells in methionine-free medium traversed G1 slowly at 38.5°C, with only 20% of the cells budded at 2.5 hr after release from the G1 arrest (Fig. 3A). Upon expression of *CLN1* through *MET3* promoter, more than 60% of *cdc48-3* cells budded at 1.5 hr after G1 release (Fig. 3A). The expression of *CLN2* in *cdc48-3* also expedited G1 progression, with ∼35% of the cells budded at 1.5 hr after G1 release (Fig. 3A). Western blots showed that the ectopically expressed Cln1 and Cln2 proteins can be detected by 30 min after induction (Fig. 3B). These results show that overexpression of either Cln1 or Cln2 protein can partially rescue the G1 delay of *cdc48-3* at high temperature and that Cln1 is more effective in driving G1 progression than Cln2 is under this condition.

### Prolonged activation of Mpk1 in *cdc48-3*

**Figure 4:**
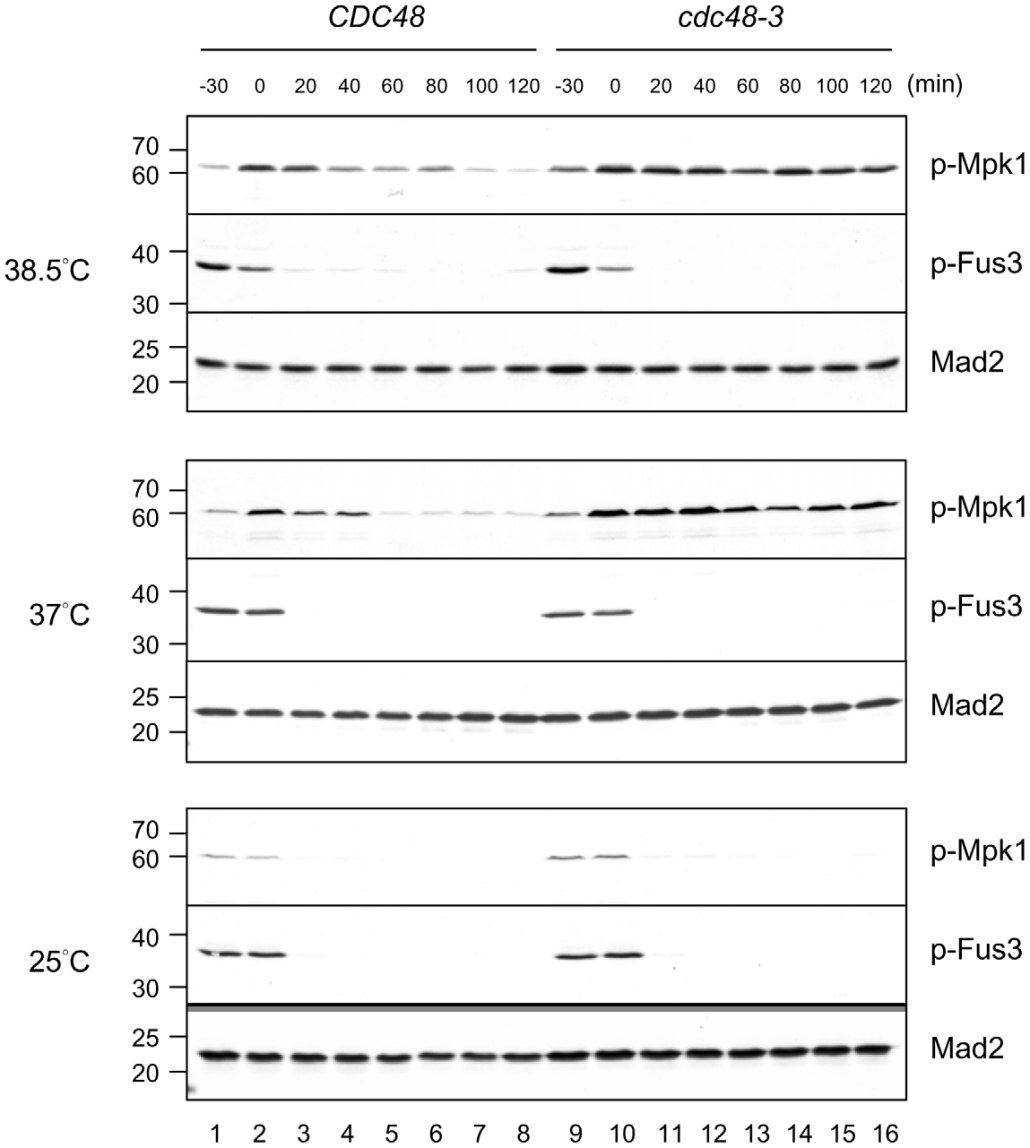
Mpk1 phosphorylation is prolonged in *cdc48-3* at high temperature. *CDC48* and *cdc48-3* cells were first arrested at G1 with α-factor at 25°C. The cells were then shifted to the temperature indicated on the left during the last 30 min of the arrest (lanes 1 and 9, immediately before temperature shift), and then released into the cell cycle at the same temperature. Samples were taken at the indicated times after the release for Western blot with anti-phospho-MAPK antibody that recognizes both phosphorylated Mpk1 and Fus3. The migration of molecular size standard is indicated on the left. Mad2 blot serves as a loading control.

Heat shock is known to transiently arrest yeast cells in G1, raising the possibility that the G1 delay of *cdc48-3* at 38.5°C may be a consequence of heat stress. We thus examined Mpk1, a MAPK family member and a component of the cell wall integrity pathway that is activated by phosphorylation in response to perturbation of the cell wall from various stress conditions including heat shock. We monitored phosphorylated Mpk1 with a phospho-MAPK antibody that recognizes several phosphorylated MAPK members. In wild-type cells arrested at G1 with α-factor, Mpk1 phosphorylation increased when the growth temperature was shifted from 25°C to 38.5°C (Fig. 4, top panel, compare lanes 1 and 2). After release from the arrest at 38.5°C, the phosphorylation remained for 20 min and gradually declined afterwards (Fig. 4, top panel). Phosphorylation of Mpk1 in *cdc48-3* was enhanced both at 25°C and upon temperature up-shift in comparison with that in the wild-type cells, and it sustained for at least 2 hr after release from α-factor arrest (Fig. 4, top panel). The level of Mpk1 phosphorylation declined faster at 37°C than that at 38.5°C (Fig. 4, middle panel). At 37°C, Mpk1 in *cdc48-3* was still phosphorylated to a higher degree than that in the wild-type cells (Fig. 4, middle panel). At 25°C, Mpk1 phosphorylation disappeared shortly after release from α-factor arrest in both wild-type and *cdc48-3* cells (Fig. 4, bottom panel). The anti-phospho-MAPK antibody also recognized phosphorylated Fus3, a mating-specific MAPK member that arrests the cell cycle through transcriptional repression of *CLN1* and *CLN2* and through posttranscriptional inhibition of Cln3 [39]. Fus3 phosphorylation was present in cells arrested with α-factor and quickly disappeared upon release from the arrest in both wild-type and *cdc48-3* cells at all temperatures tested (Fig. 4). The enhanced phosphorylation of Mpk1 in *cdc48-3* at high temperature suggests that the heat stress may be exacerbated in *cdc48-3* mutant.

**Figure 5:**
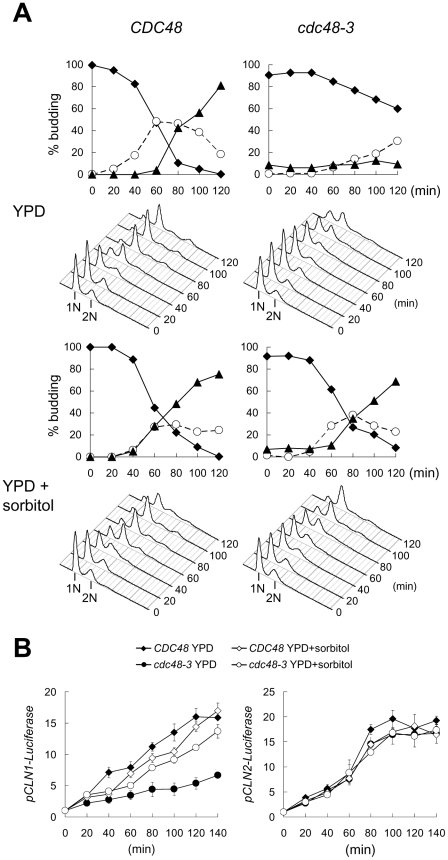
High osmolarity rescues G1 defects of *cdc48-3*. (A) *CDC48* and *cdc48-3* cells were first arrested at G1 with α-factor. The cells were shifted to 38.5°C during the last 30 min of the arrest in YPD or YPD containing 1 M sorbitol. The cells were then released in the same medium at 38.5°C, and samples were taken for budding index determination and FACS analysis. (B) *CDC48* and *cdc48-3* cells carrying luciferases under the control of *CLN1* and *CLN2* promoters were grown as described above and samples were taken at the indicated times for the measurement of luciferase activities. The plot shows the average of three measurements in fold increase and the standard deviation.

Because Mpk1 is a component of the cell wall integrity pathway, the enhanced phosphorylation of Mpk1 in *cdc48-3* indicates a defect in the cell wall at high temperature. We tested this possibility by including 1 M sorbitol in the medium to increase the osmolarity which is known to protect the cell wall and prevent cell lysis in mutants defective in the cell wall integrity pathway. Without sorbitol addition less than 40% of *cdc48-3* cells were budded at 120 min after release from G1 arrest, whereas more than 90% of the cells were budded in the presence of sorbitol (Fig. 5A). Sorbitol addition also accelerated DNA replication in *cdc48-3* at 38.5°C, with a small lag compared to the wild-type cells (Fig. 5A). In addition, reporter assays showed that sorbitol treatment increased the *CLN1* promoter activity in *cdc48-3* at 38.5°C, although the activity was still slightly lower than that in the wild-type cells (Fig. 5B). On the other hand, *CLN2* promoter activity at 38.5°C was not affected by sorbitol addition (Fig. 5B). These results show that high osmolarity can rescue the G1 delay of *cdc48-3*, which implies that *cdc48-3* was defective in maintaining the cell wall integrity during heat shock.

### Npl4 and Ufd1 are involved in G1 progression

**Figure 6:**
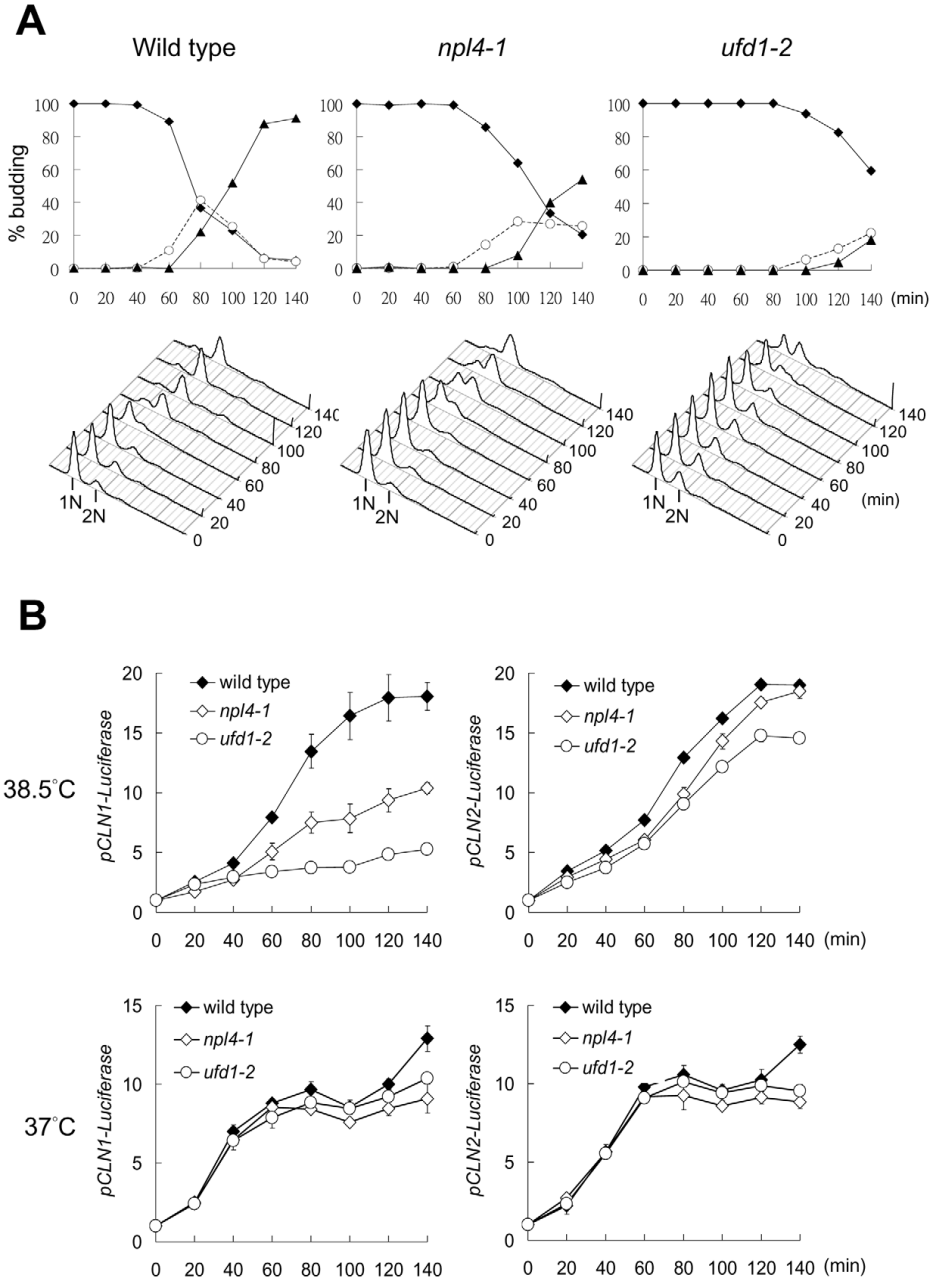
G1 progression is delayed in *npl4-1* and *ufd1-2* at high temperature. (A) Wild-type, *npl4-1*, and *ufd1-2* cells were released from G1 arrest at 38.5°C as described in Figure 1B. Budding index and FACS were analyzed after the lease. Filled diamond, no bud; open circle, small bud; filled triangle, medium/large bud. (B) Wild-type, *npl4-1*, and *ufd1-2* cells carrying luciferases under *CLN1* and *CLN2* promoters were released from G1 arrest at 38.5°C or 37°C as described in Figure 1B. Samples were taken at the indicated times after the release for the measurement of luciferase activities. The plot shows the average of three measurements in fold increase and the standard deviation.

Because Cdc48 executes its diverse functions through specific cofactors, we searched for the cofactors of Cdc48 involved in G1 progression. The known Cdc48 cofactors include Npl4-Ufd1 complex and a family of UBX domain-containing proteins. The deletion mutants of the UBX family proteins did not display specific G1 delay at high temperature (data not shown), whereas the temperature-sensitive *npl4-1* and *ufd1-2* mutants were much delayed in both budding and DNA replication upon release from α-factor arrest at 38.5°C (Fig. 6A). Similar to *cdc48-3*, the promoter activity of *CLN1*, but not *CLN2*, was reduced in *npl4-1* and *ufd1-2* at 38.5°C, but not at 37°C (Fig. 6B). These results indicate that Npl4-Ufd1 complex mediates the function of Cdc48 in G1.

**Figure 7:**
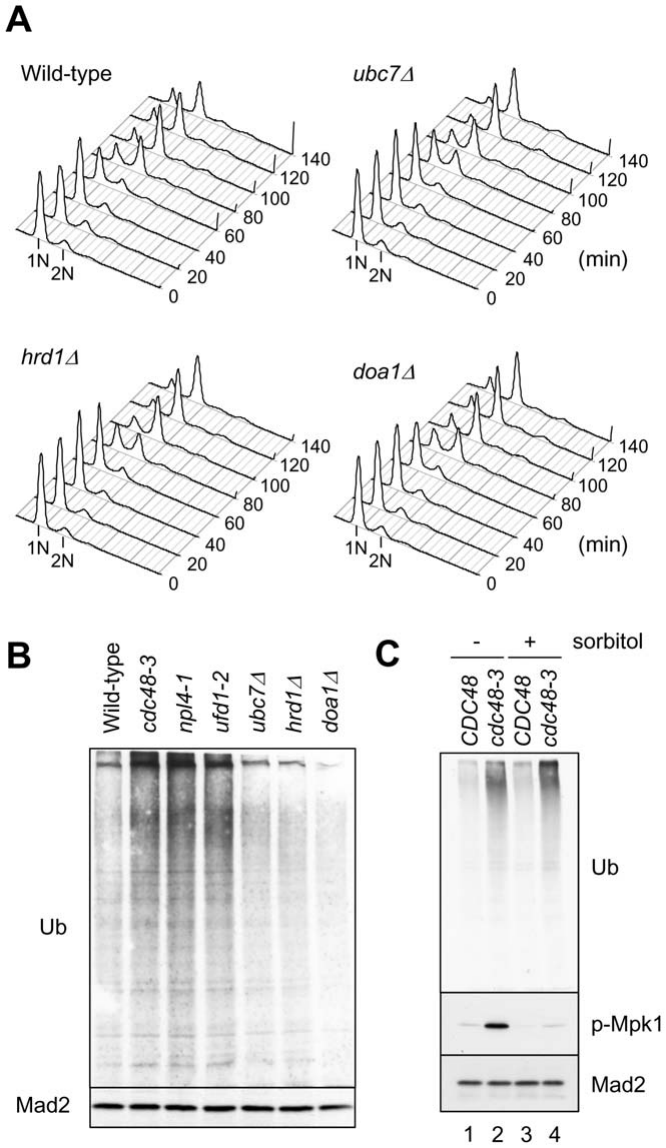
G1 defect of *cdc48-3* is independent of its ERAD function. (A) Wild-type, *ubc7Δ*, *hrd1Δ*, and *doa1Δ* cells were released from G1 arrest at 38.5°C as described in Figure 1B. Samples were taken at the indicated times for FACS analysis. (B) Wild-type or the indicated mutant cells were grown at 38.5°C for 3 hr. Cell lysates were prepared for Western blots with anti-ubiquitin (Ub) and anti-Mad2 antibodies. Mad2 serves as a loading control. (C) Wild-type (lanes 1 and 3) and *cdc48-3* (lanes 2 and 4) cells were shifted to 38.5°C for 3 hr in the presence (lanes 3 and 4) or absence (lanes 1 and 2) of 1 M sorbitol. Cell lysates were prepared for Western blots with anti-ubiquitin (Ub), anti-phospho-MAPK (p-Mpk1), and anti-Mad2 antibodies.

Cells grown at high temperature may accumulate denatured proteins that need to be folded by chaperones or be degraded by the ubiquitin-proteasome system. Because Cdc48 and Npl4-Ufd1 complex are important for ERAD, the G1 delay of *cdc48-3*, *npl4-1*, and *ufd1-2* cells at 38.5°C may be related to their ERAD function. We thus examined the deletion of two other components of the ERAD system, the ubiquitin-conjugation enzyme *UBC7* and the ubiquitin-protein ligase *HRD1* [40]. FACS analysis showed that *ubc7Δ* and *hrd1Δ* mutants had only a small delay in DNA replication during a synchronized cell cycle at 38.5°C (Fig. 7A). This result suggests that defects in ERAD do not impact on G1 progression during heat shock and that the G1 delay of *cdc48-3*, *npl4-1*, and *ufd1-2* mutants at 38.5°C is probably independent of their functions in ERAD. To determine if the G1 delay involves other aspects of protein degradation, we examined the deletion of *DOA1*, a ubiquitin-binding protein that bridges Cdc48 to its substrates for protein degradation [41]. FACS analysis showed that *doa1Δ* behaved essentially the same as the wild-type cells at 38.5°C (Fig. 7A). In addition, *cdc48-3*, *npl4-1*, and *ufd1-2* cells at 38.5°C contained more ubiquitin-cojugated proteins than did wild-type cells, whereas *ubc7Δ*, *hrd1Δ*, and *doa1Δ* cells did not (Fig. 7B). We also analyzed additional ERAD components, including ubiquitin ligase Doa10 [40], the cytosolic Hsp70 chaperone Ssa1 involved in ERAD of a membrane protein [42], and Ubx2 that recruits Cdc48 to ERAD ubiquitin ligase [40]. Because *ubx2Δ* deletion mutant grew very slowly in our strain background, we conditionally controlled the expression of *UBX2* with a galactose-inducible promoter that can be suppressed with glucose. Similar to *ubc7Δ* and *hrd1Δ* cells, *doa10Δ*, *ssa1Δ*, and Ubx2-depleted cells did not accumulate ubiquitin conjugates (Fig. S1). Furthermore, additional mutations in the ERAD system did not abolish the ubiquitin conjugates in *cdc48-3* (Fig. S1), indicating that the proteins were ubiquitylated by an ERAD-independent mechanism.

The accumulation of ubiquitylated proteins may lead to Mpk1 activation and G1 arrest in *cdc48-3*. To test this possibility, we examined the effect of adding 1 M sorbitol to the medium during temperature shift to 38.5°C, which suppressed G1 delay and enhanced *CLN1* promoter in *cdc48-3* (Fig. 5). Figure 7C shows that addition of sorbitol greatly reduced the level of phosphorylated Mpk1 in both wild-type and *cdc48-3* cells without an obvious effect on the level of ubiquitin conjugates in *cdc48-3* cells. Therefore, accumulation of ubiquitylated proteins per se does not cause Mpk1 activation and G1 arrest in *cdc48-3*.

**Figure 8:**
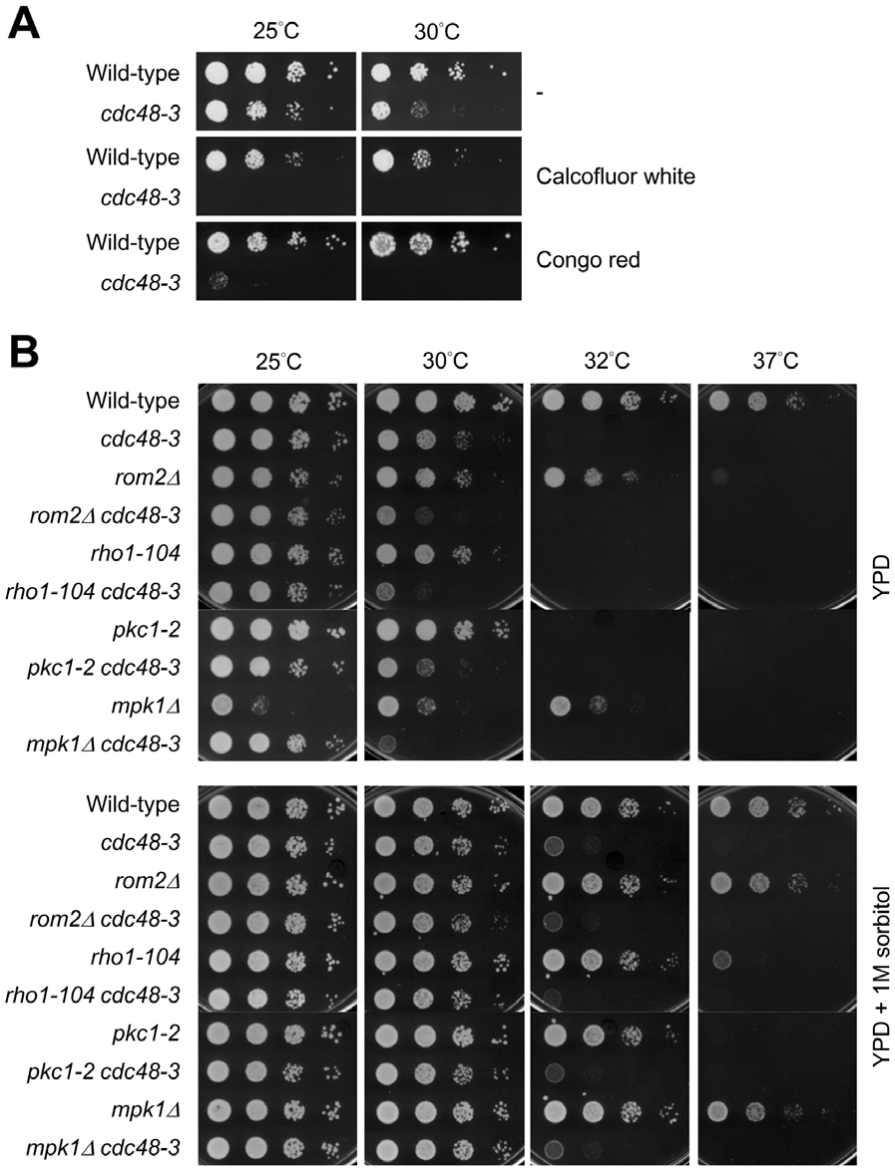
The cell wall is defective in *cdc48-3*. (A) Wild-type and *cdc48-3* cells were spotted in 10-fold serial dilutions from left to right on YPD (top), YPD containing 25 µg/ml Calcofluor white (middle) or 100 µg/ml Congo red (bottom). Plates were incubated at 25°C or 30°C and photographed after 1–3 days. (B) Wild-type and the indicated mutant strains were spotted in 10-fold serial dilutions from left to right on YPD (upper) or YPD containing 1 M sorbitol (lower). Plates were incubated at temperatures indicated on the top.

Our results of sorbitol treatment suggest that a cell wall defect is likely the direct cause of Mpk1 phosphorylation and G1 delay in *cdc48-3*. Thus, we tested the sensitivity of *cdc48-3* to two cell wall perturbing agents, Calcofluor white and Congo red. Figure 8A shows that, unlike wild-type cells, *cdc48-3* cells were unable to grow on YPD plates containing either Calcofluor white or Congo red even at 25°C or 30°C. This result suggests that the cell wall is defective in *cdc48-3*.

To further link *cdc48-3* to cell wall defect, we examined genetic interaction between *cdc48-3* and components of the cell wall integrity pathway, including Mpk1, Pkc1 (the upstream kinase of Mpk1), Rho1 (a G-protein and a regulator for Pkc1), and Rom2 (GDP/GTP exchange factor for Rho1). The single mutants of these components were growth-defective at elevated temperatures, which can be suppressed by the addition of 1 M sorbitol in the medium up to 35°C (Fig. 8B and data not shown). Their double mutants with *cdc48-3* showed more severe growth phenotype than did the single mutants (Fig. 8B). In the presence of sorbitol the double mutants can grow at 30°C but behaved as *cdc48-3* alone at 32°C (Fig. 8B). The synthetic phenotype in the double mutants and its suppression by sorbitol suggest that failure to activate the cell wall integrity pathway in *cdc48-3* compromises cell viability and that *cdc48-3* normally activates this signaling pathway to repair its cell wall defect.

## Discussion

Mild heat shock is known to transiently arrest yeast cells at G1 [30]. Herein we report that mutations in Cdc48 and its cofactors Npl4 and Ufd1 prolong the G1 delay in the budding yeast *Saccharomyces cerevisiae* at 38.5°C. This delay is due to a low *CLN1*, but not *CLN2*, promoter activity. Several lines of evidence support that the G1 delay of *cdc48-3* at 38.5°C is a consequence of cell wall defect. First, phosphorylation of Mpk1, a MAPK family member important for the cell wall integrity pathway, is increased in *cdc48-3* at 38.5°C. Second, the *CLN1* promoter activity and the G1 delay in *cdc48-3* are rescued by an increase of osmolarity in the medium to protect the cell wall. Furthermore, *cdc48-3* is hypersensitive to cell wall perturbing agents and is synthetic-sick with mutations in the cell wall integrity signaling pathway. Our study suggests that Cdc48 is important for cell wall integrity. The cell wall defect in *cdc48-3* is probably exacerbated at high temperature (38.5°C) to a degree that over-activates the cell wall integrity pathway and delays G1 progression.

We show that G1 cyclins *CLN1* and *CLN2* are differentially regulated in *cdc48-3* at 38.5°C. The activity of *CLN1* promoter is lower in *cdc48-3* mutant at 38.5°C than that in the wild-type cells, whereas the *CLN2* promoter activities are comparable in both strains. Both *CLN1* and *CLN2* promoters are activated by Swi4/Swi6 (SBF) and Mbp1/Swi6 (MBF) complexes that recognize multiple SCB and MCB sequences, respectively, in the upstream regions of *CLN1* and *CLN*2 [43]. Thus, *CLN1* and *CLN2* are known to be regulated similarly during the cell cycle. Our study provides a rare example that these genes can be regulated differently. Heat stress is known to induce the transcription repressor Xbp1 that shares homology with Swi4 and Mbp1 in the DNA-binding domain [44]. Despite the structural similarity, the DNA recognition sequence of Xbp1 is distinct from Swi4/Swi6 and Mbp1/Swi6 binding sites. The binding motif for Xbp1 is present in *CLN1* promoter, and overexpression of Xbp1 can repress the expression of G1 cyclins and lengthen G1. We have found that deletion of *XBP1* gene in *cdc48-3* promoted budding at 38.5°C, compared to *cdc48-3* alone (Fig. S2), suggesting that Xbp1 contributes to the suppression of *CLN1* promoter in *cdc48-3*. How heat stress may control the activity of Xbp1 remains to be determined. Furthermore, the levels of Swi4 and Swi6 proteins are similar in wild-type and *cdc48-3* cells at 38.5°C (Fig. S3). However, we cannot exclude the possibility that these transcription activators are modified differently in response to heat stress in *cdc48-3* cells and that *CLN1* promoter may be more sensitive to the small alteration of these proteins.

A role for Cdc48 in G1 has been suggested previously [14]. By using a temperature-sensitive degron-tagged *cdc48-td* mutant, it has been shown that Cdc48 is required for the execution of Start commitment point in *Saccharomyces cerevisiae* by degrading the G1-cyclin-dependent kinase inhibitor Far1 [45]. In *cdc48-3* mutant Far1 is still degraded with kinetics similar to that in the wild-type cells at 38.5°C (Fig. S3), indicating that the G1 delay of *cdc48-3* is not due to a defect in the degradation of Far1. Together, studies with different *cdc48* mutant alleles reveal that Cdc48 is important for G1 progression during a normal cell cycle and under heat stress through different mechanisms.

Heat shock is known to transiently arrest cells before Start by repressing transcription of *CLN1* and *CLN2* [46]. Deletion of any G1 cyclin genes has no significant effect on the transient arrest from heat shock at 37°C [46]. In our study we found that G1 progression is further delayed in *cdc48-3* at 38.5°C, even though *CLN2* expression is similar to that of wild-type cells. It is probable that cells require more G1 cyclin activity to recover from the heat stress incurred at a higher temperature.

Cdc48 is best known for its function in ERAD that is one of the protein quality control systems [47]. Secretory and membrane proteins are normally synthesized and folded in the ER. Misfolded or unassembled proteins are retained in the ER and subsequently degraded via ERAD. Heat shock may cause accumulation of misfolded or aggregated proteins in the ER that need to be removed through the ERAD pathway. Indeed, we found that *cdc48-3*, *npl4-1*, and *ufd1-2* mutants accumulated higher levels of ubiquitin conjugates than did the wild-type cells at 38.5°C (Fig. 7B). Subcellular fractionation shows that the ubiquitin conjugates were enriched in the ER fractions (Fig. S4). Interestingly, mutations in other ERAD components did not abolish the accumulation of ubiquitylated proteins in *cdc48-3* (Fig. S1). Thus, Cdc48-Npl4-Ufd1 is also required for degradation of ER proteins that are ubiquitylated by ERAD-independent pathway. Furthermore, deletion of ERAD components *UBC7* and *HRD1* has no significant effect on the G1 progression at 38.5°C, suggesting that a defect in ERAD system does not cause G1 arrest in response to heat stress. Therefore, the G1 delay of *cdc48-3*, *npl4-1*, and *ufd1-2* mutants at 38.5°C is independent of their ERAD function. However, we cannot exclude the possibility that there may be other unknown or redundant ERAD components that function together with Cdc48-Npl4-Ufd1 to remove the ubiquitylated proteins from the ER.

We observe enhanced phosphorylation of Mpk1, a MAPK family member downstream of Pkc1, in *cdc48-3* mutant at 38.5°C. This pathway is activated by hypotonic shock or by heat stress [48], and the activation is sustained during growth at a high temperature [33]. Because Pkc1-regulated signaling pathway is known to detect and respond to weakness in the cell wall, the sustained phosphorylation of Mpk1 in *cdc48-3* suggests that the defect in the cell wall or the plasma membrane is not repaired. That the addition of sorbitol restores cell growth without affecting the overall ubiquitylation level (Figs. 5 and 7C) indicates that the G1 defect of *cdc48-3* is caused by cell wall defect, rather than the accumulation of denatured proteins per se. The sensitivity of *cdc48-3* to cell wall perturbing agents at permissive temperature indicates a cell wall defect that is likely exacerbated at high temperature. In addition, Mpk1 phosphorylation is also increased in the cold-sensitive *cdc48-1* mutant at 14°C compared to that in the wild-type cells (Fig. S5A), which is consistent with the notion that Cdc48 is required for cells wall integrity. Mpk1 is increasingly phosphorylated with elevated temperatures and the levels are similar between the wild-type and *cdc48-1* cells (Fig. S5A), showing that the cold-sensitive *cdc48-1* mutant has normal response to heat shock. On the other hand, Mpk1 is phosphorylated to a higher degree in *cdc48-3* than in the wild-type cells at both 37° and 38.5°C (Fig. 4 and S5B). We believe that the heat-induced Mpk1 activation in wild-type cells elicits cell wall repair and transient G1 arrest, whereas overactivation of Mpk1 in *cdc48-3* caused by failure to repair the cell wall prolongs G1 arrest.

Heat shock is known to create cell wall stress that activates the cell wall integrity pathway, leading to phosphorylation and activation of transcription factor Rlm1 by Mpk1 [37]. Rlm1 induces expression of many genes implicated in cell wall biogenesis [49]. Mutants in this pathway are deficient in cell wall construction, leading to cell lysis at elevated temperatures. The enzymes for cell wall biogenesis mostly reside in the plasma membrane or the cell wall [50] and are synthesized and modified in ER. It has been shown that proteins synthesized during heat shock are prone to denaturation and are rapidly degraded through Cdc48-Npl4-Ufd1, independently of ERAD [51]. The accumulation of ubiquitylated proteins in the ER of *cdc48-3* cells (Fig. S4) suggests a possibility that some of the newly synthesized enzymes for cell wall biogenesis may be denatured and ubiquitylated in the ER during heat shock. Inability to efficiently degrade these proteins in *cdc48-3* cells may perturb repair of the cell wall and sustain cell wall integrity pathway, leading to G1 arrest. This effect is specific to certain targets of Cdc48, rather than a general inhibition of protein degradation, because the level of Mpk1 phosphorylation in the proteasome mutants *rpt2RF* and *rpt5S* is comparable to that in the wild type at 38.5°C (Fig. S5C). In addition, these proteasome mutants are not sensitive to chemicals that perturb the cell wall (data not shown). These results suggest that defects in proteasomal degradation and accumulation of polyubiquitylated protein per se do not cause cell wall defect or Mpk1 overactivation. It is probable that these proteasome mutants do not accumulate polyubiquitylated proteins in the ER to perturb the synthesis or maturation of cell wall repair enzymes. The direct targets of Cdc48 in the cell wall biogenesis pathway remain to be determined in the future.

## Materials and Methods

### Growth of yeast

YEPD medium contained 1% yeast extract, 2% bacto-peptone, and 2% glucose. Complete synthetic medium contained 0.67% yeast nitrogen base without amino acids (YNB), 1× complete supplement mixture (CSM) (Bio 101), and 2% glucose. For induction from the *MET3* promoter, 1× CSM was replaced by 1× CSM without methionine. To arrest cells at G1, α-factor (Sigma) was added at 1 µg/ml from a 10 mg/ml stock in DMSO and the cells were grown at 25°C for 3 hr. Cells were then shifted to the indicated temperature for 30 min. To release from G1 arrest, the cells were washed 3 times with warm water and then resuspended in pre-warmed medium at the indicated temperature.

### Construction of plasmids and yeast strains

Table 1 lists the yeast strains used in this work. All strains are derivatives of W303, except *cdc48-1* [1], *rpt2RF*, *and rpt5S* [52] that were used in Figure S5. *npl4-1, ufd1-2,* and *rho1-104* mutants were backcrossed four times to W303. Gene deletions, epitope tagging, and introduction of *pGAL* to *UBX2* were generated by PCR-mediated integration [53]. For the reporter constructs, the promoter regions (1000 bp upstream of the start codon) of *CLN1* and *CLN2* were amplified by PCR from yeast genomic DNA and cloned at *Sac*I and *Xba*I sites upstream of *Renilla reniformis (RLUC)* and *Pyrophorus plagiophthalamus* (*spLUC)* Luciferase, respectively, in pRS416. *pCLN1-RLUC* and *pCLN2-spLUC* regions were then removed with *Sac*I and *Sma*I and cloned into the cognate sites in vectors pRS404 and pRS405, respectively. pRS404-*pCLN1-RLUC* and pRS405-*pCLN2-spLUC* were linearlized with *Bsu36*I and *Xcm*I, respectively, to integrate into yeast genome at the selection markers.

**Table 1:** Yeast strains used in this study.

Strains	Genotype
W303	*ade2-1 can1-100 his3-11,15 leu2-3,112 trp1-1 ura3-1 bar1Δ MAT* ***a***
RHC677	*cdc48-3::HIS3*
RHC2078	*pCLN1-R-Luc::TRP1 pCLN2-sp-Luc::LEU2*
RHC2079	*pCLN1-R-Luc::TRP1 pCLN2-sp-Luc::LEU2 cdc48-3::HIS3*
RHC1445	*pMET3-2myc-CLN1::LEU2*
RHC1446	*pMET3-2myc-CLN1::LEU2 cdc48-3::HIS3*
RHC1553	*pMET3-2myc-CLN2:: LEU2*
RHC1554	*pMET3-2myc-CLN2::LEU2 cdc48-3:: HIS3*
RHC1122	*ufd1-2*
RHC1126	*npl4-1*
RHC2080	*pCLN1-R-Luc::TRP1 pCLN2-sp-Luc::LEU2 npl4-1*
RHC2081	*pCLN1-R-Luc::TRP1 pCLN2-sp-Luc::LEU2 ufd1-2*
RHC1764	*rom2Δ::URA3*
RHC1767	*rom2Δ::URA3 cdc48-3::HIS3*
RHC1760	*rho1-104*
RHC1762	*rho1-104 cdc48-3::HIS3*
RHC1769	*pkc1-2*[Ycp50] *pkc1Δ::LEU2*
RHC1772	*pkc1-2*[Ycp50] *pkc1Δ::LEU2 cdc48-3::HIS3*
RHC2210	*mpk1Δ::HYG*
RHC2211	*mpk1Δ::HYG cdc48-3::HIS3*
RHC1740	*SWI4-3HA::TRP1*
RHC1741	*SWI4-3HA::TRP1 cdc48-3::HIS3*
RHC1742	*SWI6-3HA::TRP1*
RHC1743	*SWI6-3HA::TRP1 cdc48-3::HIS3*
RHC1746	*FAR1-3HA::KanMX6*
RHC1747	*FAR1-3HA::KanMX6 cdc48-3::HIS3*
RHC1801	*CLN1-3HA::KanMX6 xbp1Δ::TRP1*
RHC1802	*CLN1-3HA::KanMX6 xbp1Δ::TRP1 cdc48-3::HIS3*
RHC1726	*ubc7Δ::KanMX6*
RHC1729	*ubc7Δ::KanMX6 cdc48-3::HIS3*
RHC1727	*hrd1Δ:: KanMX6*
RHC1730	*hrd1Δ:: KanMX6 cdc48-3::HIS3*
RHC1728	*doa1Δ:: KanMX6*
RHC2118	*doa10Δ::HYG*
RHC2119	*doa10Δ::HYG cdc48-3::HIS3*
RHC2120	*ssa1Δ::HYG*
RHC2121	*ssa1Δ::HYG cdc48-3::HIS3*
RHC1531	*pGAL-UBX2::TRP1*
RHC1834	*pGAL-UBX2::TRP1 cdc48-3::HIS3*

### Western blot

1.5 ml of yeast culture at OD_600_ ∼1 was collected and washed once with cold TE (10 mM Tris [pH 8.0], 1 mM EDTA). Cell pellets were frozen at −80°C if not used immediately. Proteins were extracted by bead-beating the cell pellet in 60–80 µl lysis buffer (10 mM potassium phosphate [pH 7.2], 1 mM EDTA, 5 mM EGTA, 50 mM β-glycerophosphate, 1 mM sodium vanadate, 10 mM MgCl_2_, 0.5% Triton X-100, 0.1% sodium deoxycholate, 0.1% SDS, 1 mM PMSF, 0.5 mM DTT, 10 µg/ml each of leupeptin, pepstatin and chymostatin) with Zirconia beads at 4°C for 1 min. Samples were then centrifuged at 14,000 rpm for 5 min at 4°C, and the supernatants were taken as yeast cell lysates. For Western blot with anti-ubiquitin antibody, the cell pellets were bead-beat in RIPA (10 mM Tris [pH 7.2], 150 mM NaCl, 1% sodium deoxycholate, 1% Triton X-100, 0.1% SDS, 1 mM sodium vanadate) containing 10 mM N-Ethylmaleimide, 10 µg/ml each of leupeptin, pepstatin and chymostatin, 1 mM PMSF, and 1× protease inhibitor cocktail (Roche). Protein concentration was determined with DC protein assay kit (Biorad), and then normalized with lysis or RIPA buffer. Proteins were resolved by SDS-PAGE and transferred to nitrocellulose membrane. The membrane was first pre-blocked with blocking solution (PBS containing 2% BSA, 0.2% Tween-20, 0.05% sodium azide) for 1 hr at room temperature, followed by incubation with antibodies in blocking solution for 2 hr at room temperature. Antibody dilution: anti-myc (9E10, Covance, 1∶500), anti-HA (16B12, Covance, 1∶500), anti-Mad2 (1∶2000) [54], anti-phospho-MAPK (9101, Cell Signaling, 1∶1000), anti-ubiquitin (P4D1, Covance, 1∶500), anti-Pma1 (1∶5000), and anti-Sec61 (1∶5000). The latter two antibodies were provided by Dr. Chao-Wen Wang (IPMS, Academia Sinica).

### FACS analysis

1 ml of yeast cells were pelleted and resuspended in ice-cold fix solution (40% ethanol, 0.1 M sorbitol, 5 mM EDTA, 5 mM sodium azide). Samples were temporarily frozen at −80°C. Cells were then washed with PBS plus 0.5% Triton X-100, and incubated with 100 µg/ml of RNaseA in 50 mM Tris-Cl (pH 8.0) overnight at 37°C. Cells were then resuspended in 100–200 µl of Sytox Green (Invitrogen, 1∶400 in 38 mM sodium citrate) and briefly sonicated. Samples were diluted in 1 ml PBS and analyzed by FACSCalibur flow cytometer (BD Biosciences).

### Luciferase assay

Yeast cells carrying *pCLN1-RLUC* and *pCLN2-spLUC* were grown to mid-log phase and arrested at G1 stage by α-factor. The cells were shifted to 37°C or 38.5°C for 30 min before release from the arrest at the same temperature. At each time point, OD_600_ of the culture was measured and another 1.5 ml of yeast cells were collected. The cells were washed with cold PBS and frozen at −80°C until use. Cell pellets were resuspended to 65 µl in PBS, and 20 µl of the sample was used for each luminescence measurement with Vector3 luminometer plus autoinjector (Perkin Elmer). Through the autoinjector, 100 µl of 1 µM coelenterazine (Promega) and D-Luciferin (Sigma) was added to the sample for *R.* Luciferase and *sp* Luciferase, respectively, with 5-sec equilibration and 10-sec integration time for the measurement of luminescence. The results were normalized with OD_600_.

### Subcellular fractionation

Subcellular fractionation was performed as described [55]. Briefly, 80 OD_600_ of cells were disrupted by bead-beating in STE buffer (10 mM Tris-HCl [pH 7.6], 10 mM EDTA, 10% [wt/wt] sucrose, protease inhibitor cocktail [Roche], 1 mM PMSF, 10 mM N-Ethylmaleimide, 10 µg/ml each of leupeptin, pepstatin and chymostatin). Unbroken cells were pelleted by centrifugation at 300×*g* for 2 min at 4°C. Total lysate from the supernatant was then subjected to centrifugation at 100,000×*g* for 60 min at 4°C (TLS55 rotor, Beckman, Optima Max centrifuge). The resulting supernatant and pellet represent cytosol and membrane fractions, respectively. The membrane pellet was then resuspended in STE buffer and layered on top of a continuous gradient composed of 20–60% (wt/wt) sucrose in 5 ml of TE buffer (10 mM Tris-HCl [pH 7.6], 10 mM EDTA). After centrifugation at 100,000×*g* for 18 hr at 4°C (MLS50.1 rotor, Beckman, Optima Max centrifuge), fractions of 356 µl were manually collected from top to bottom of the gradient. Proteins were resolved by SDS-PAGE for Western blot analysis.

## Supporting Information

Figure S1 **Accumulation of ubiquitin conjugates in** ***cdc48-3*** **is independent of ERAD components.** Wild-type and the indicated mutant strains were grown to mid-log phase and shifted to 38.5°C for 3 hr. *pGAL-UBX2* and *pGAL-UBX2 cdc48-3* were first grown in YEP containing galactose to mid-log phase and then changed to YPD to suppress Ubx2 expression for 2 hr before shifting to 38.5°C for 3 hr. Cell lysates were prepared for Western blots with anti-ubiquitin (Ub) and anti-Mad2 antibodies. Mad2 serves as a loading control.
(TIF)

Figure S2 **Deletion of** ***XBP1*** **partially rescues budding defect of** ***cdc48-3*** **at 38.5°C.** Wild type, *cdc48-3*, *xbp1Δ*, and *xbp1Δ cdc48-3* cells were grown as described in Figure 1B and their budding index at the indicated times during the cell cycle entry were determined. Filled diamond, no bud; open circle, small bud; filled triangle, medium/large bud.
(TIF)

Figure S3 **The protein levels of Swi4, Swi6, and Far1 are unaffected by temperature up-shift in** ***cdc48-3*** **.** Swi4, Swi6, and Far1 were tagged at the carboxyl-terminus with 3HA at the chromosomal loci in *CDC48* and *cdc48-3* cells. The cells were arrested at G1 with α-factor and released into the cell cycle at 38.5°C as described in Figure 1B. Samples were taken at the indicated times after the release for Western blot with anti-HA antibody. Asterisks denote cross-reacting proteins. The migration of molecular size standard is indicated on the left.
(TIF)

Figure S4 **Ubiquitin conjugates are enriched in the ER fraction of** ***cdc48-3*** **.** *CDC48* and *cdc48-3* cells were grown to mid-log phase and shifted to 38.5°C for 3 hr. Total cell lysates (lanes 1 and 2) were prepared and then separated into cytosol (lanes 3 and 4) and membrane (lanes 5 and 6) fractions. The membrane fractions were further fractionated by continuous 20-60% (wt/wt) sucrose gradient (lanes 7-20, *CDC48*; lanes 21-34, *cdc48-3*). The fraction numbers from top to bottom of the gradient are indicated. Equivalent to 1/500 of the total lysate, cytosol, and membrane fractions as well as 1/100 of each fraction from the sucrose gradient were subjected to Western blot analysis with antibodies against ubiquitin (Ub), ER protein Sec61, and plasma membrane protein Pma1.
(TIF)

Figure S5 **Phosphorylation of Mpk1 is enhanced in** ***cdc48*** **mutants, but not in proteasome mutants.** Cells of indicated genotypes were first grown at 25°C and then shifted to 14°C for 2 days or other indicated temperatures for 3 hr. Cell lysates were prepared for Western blots with anti-phospho-Mpk1 and anti-Mad2 antibodies. Mad2 serves as a loading control.
(TIF)
