# Supplementary figures and images for: Cdc48 and Cofactors Npl4-Ufd1 Are Important for G1 Progression during Heat Stress by Maintaining Cell Wall Integrity in Saccharomyces cerevisiae
Source: PLoS One. 2011 Apr 19;6(4):e18988. doi: 10.1371/journal.pone.0018988 (PMC3079750; doi:10.1371/journal.pone.0018988)

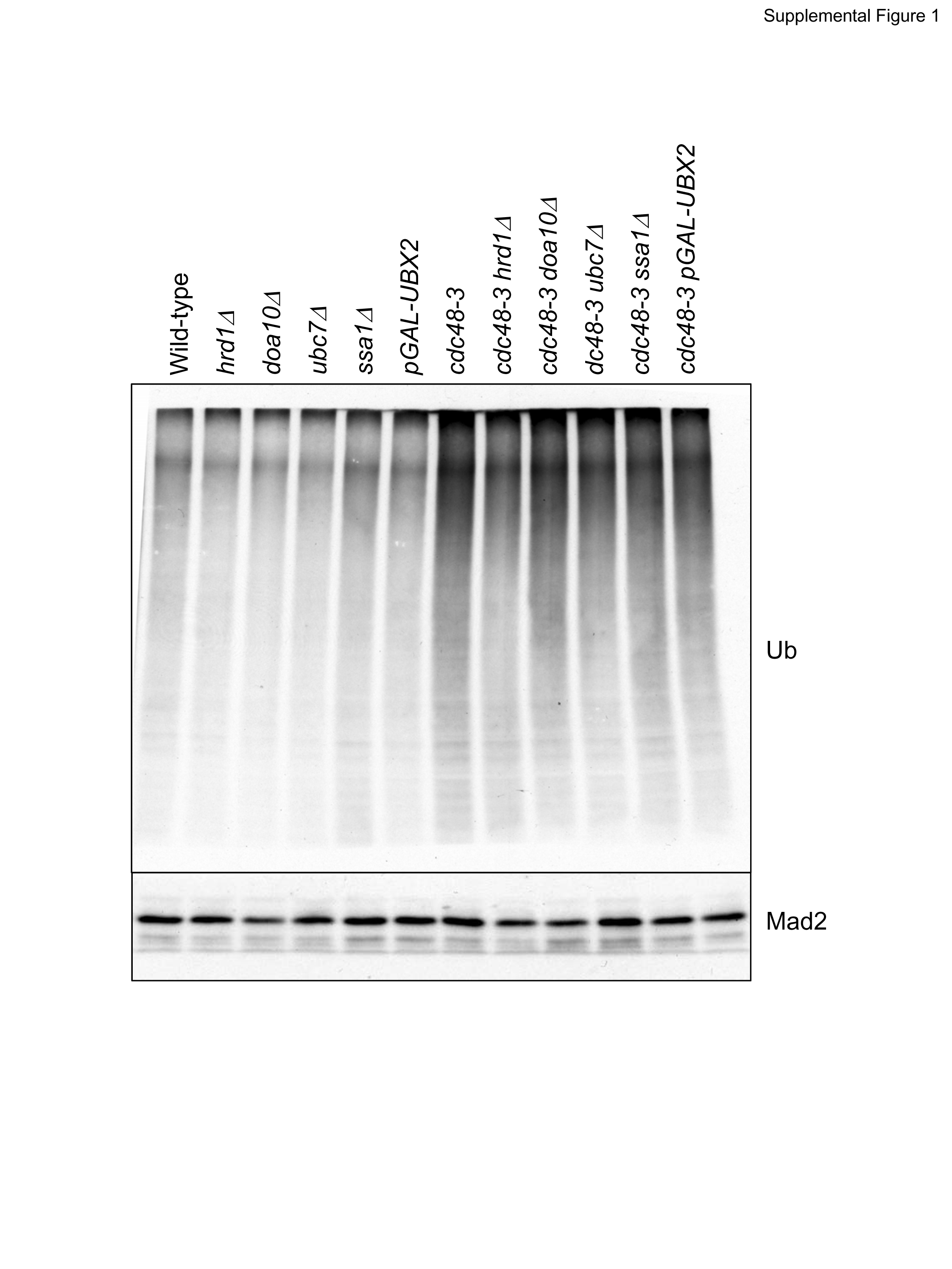

Supplement: Figure S1 — Accumulation of ubiquitin conjugates in cdc48-3 is independent of ERAD components. Wild-type and the indicated mutant strains were grown to mid-log phase and shifted to 38.5°C for 3 hr. pGAL-UBX2 and pGAL-UBX2 cdc48-3 were first grown in YEP containing galactose to mid-log phase and then changed to YPD to suppress Ubx2 expression for 2 hr before shifting to 38.5°C for 3 hr. Cell lysates were prepared for Western blots with anti-ubiquitin (Ub) and anti-Mad2 antibodies. Mad2 serves as a loading control. (TIF) [file pone.0018988.s001.tif]

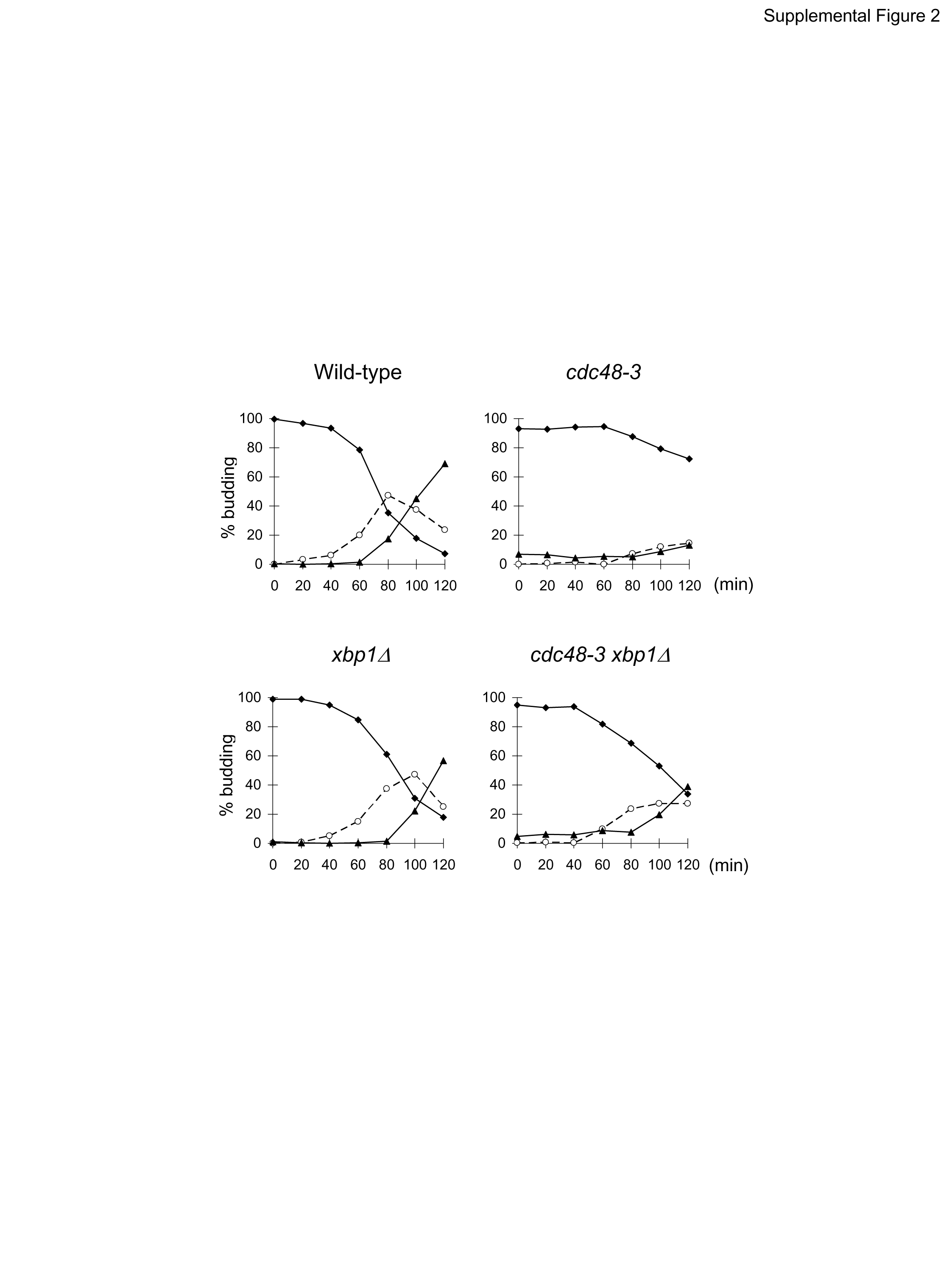

Supplement: Figure S2 — Deletion of XBP1 partially rescues budding defect of cdc48-3 at 38.5°C. Wild type, cdc48-3, xbp1Δ, and xbp1Δ cdc48-3 cells were grown as described in Figure 1B and their budding index at the indicated times during the cell cycle entry were determined. Filled diamond, no bud; open circle, small bud; filled triangle, medium/large bud. (TIF) [file pone.0018988.s002.tif]

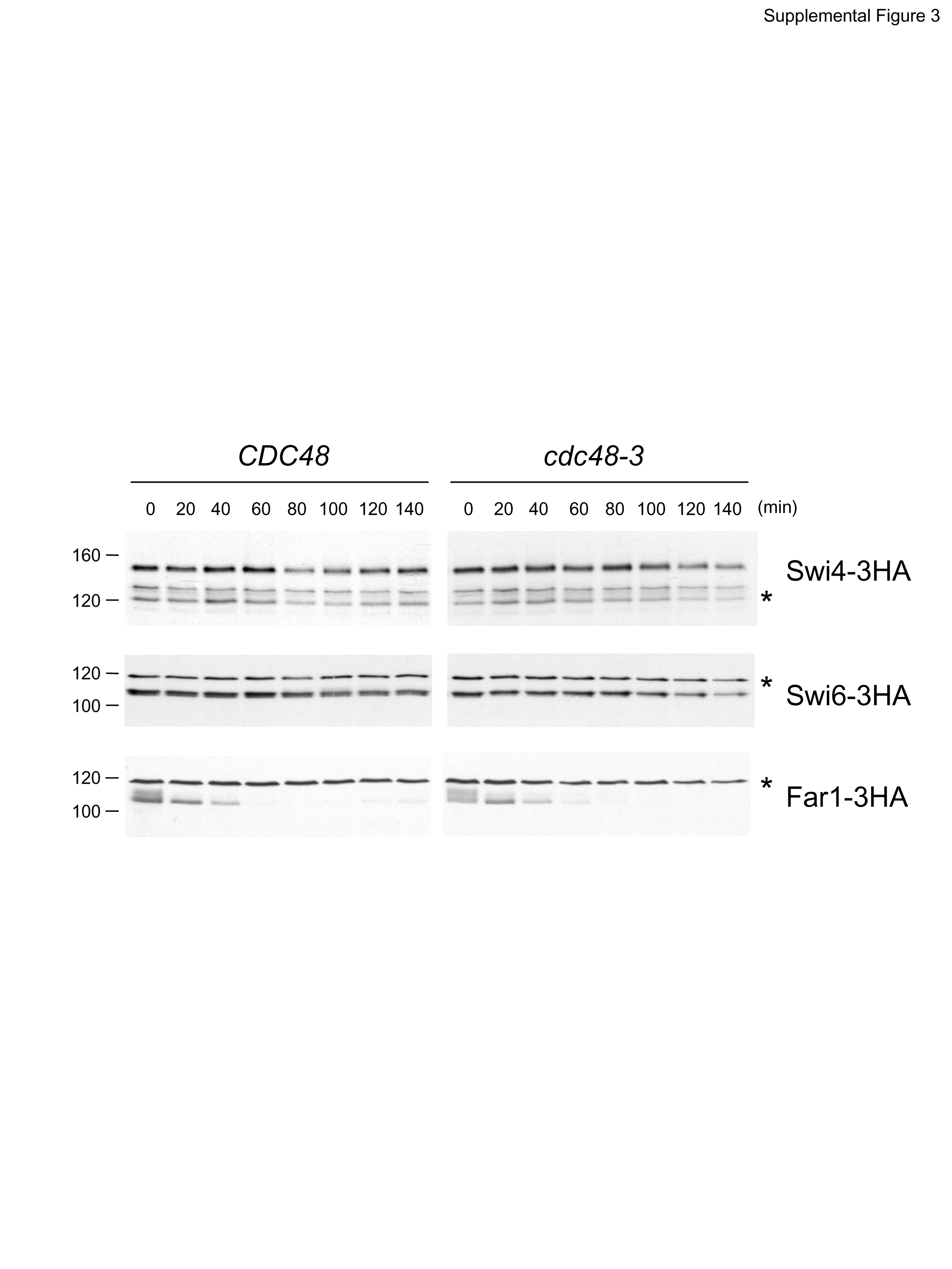

Supplement: Figure S3 — The protein levels of Swi4, Swi6, and Far1 are unaffected by temperature up-shift in cdc48-3 . Swi4, Swi6, and Far1 were tagged at the carboxyl-terminus with 3HA at the chromosomal loci in CDC48 and cdc48-3 cells. The cells were arrested at G1 with α-factor and released into the cell cycle at 38.5°C as described in Figure 1B. Samples were taken at the indicated times after the release for Western blot with anti-HA antibody. Asterisks denote cross-reacting proteins. The migration of molecular size standard is indicated on the left. (TIF) [file pone.0018988.s003.tif]

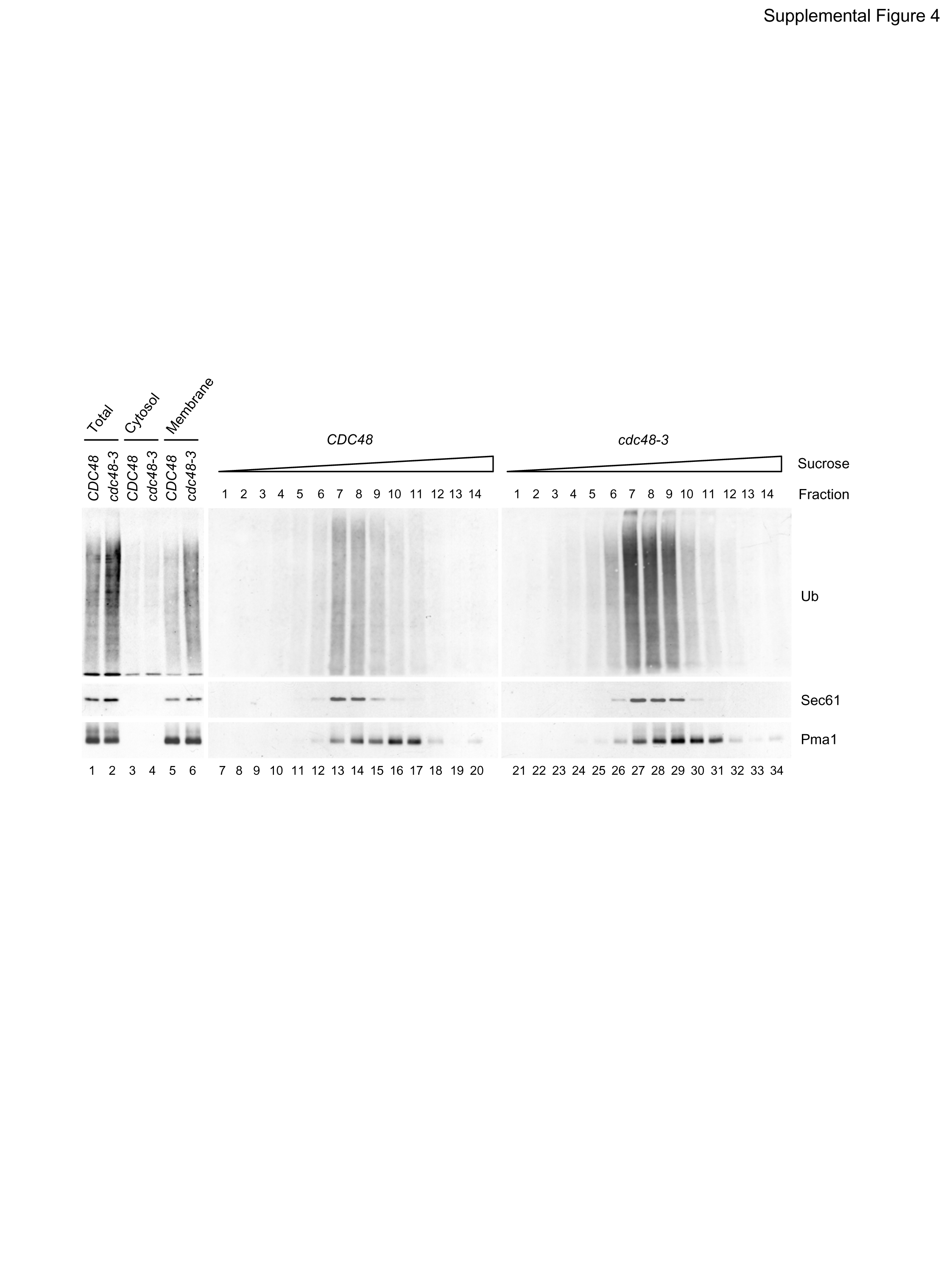

Supplement: Figure S4 — Ubiquitin conjugates are enriched in the ER fraction of cdc48-3 . CDC48 and cdc48-3 cells were grown to mid-log phase and shifted to 38.5°C for 3 hr. Total cell lysates (lanes 1 and 2) were prepared and then separated into cytosol (lanes 3 and 4) and membrane (lanes 5 and 6) fractions. The membrane fractions were further fractionated by continuous 20-60% (wt/wt) sucrose gradient (lanes 7-20, CDC48; lanes 21-34, cdc48-3). The fraction numbers from top to bottom of the gradient are indicated. Equivalent to 1/500 of the total lysate, cytosol, and membrane fractions as well as 1/100 of each fraction from the sucrose gradient were subjected to Western blot analysis with antibodies against ubiquitin (Ub), ER protein Sec61, and plasma membrane protein Pma1. (TIF) [file pone.0018988.s004.tif]

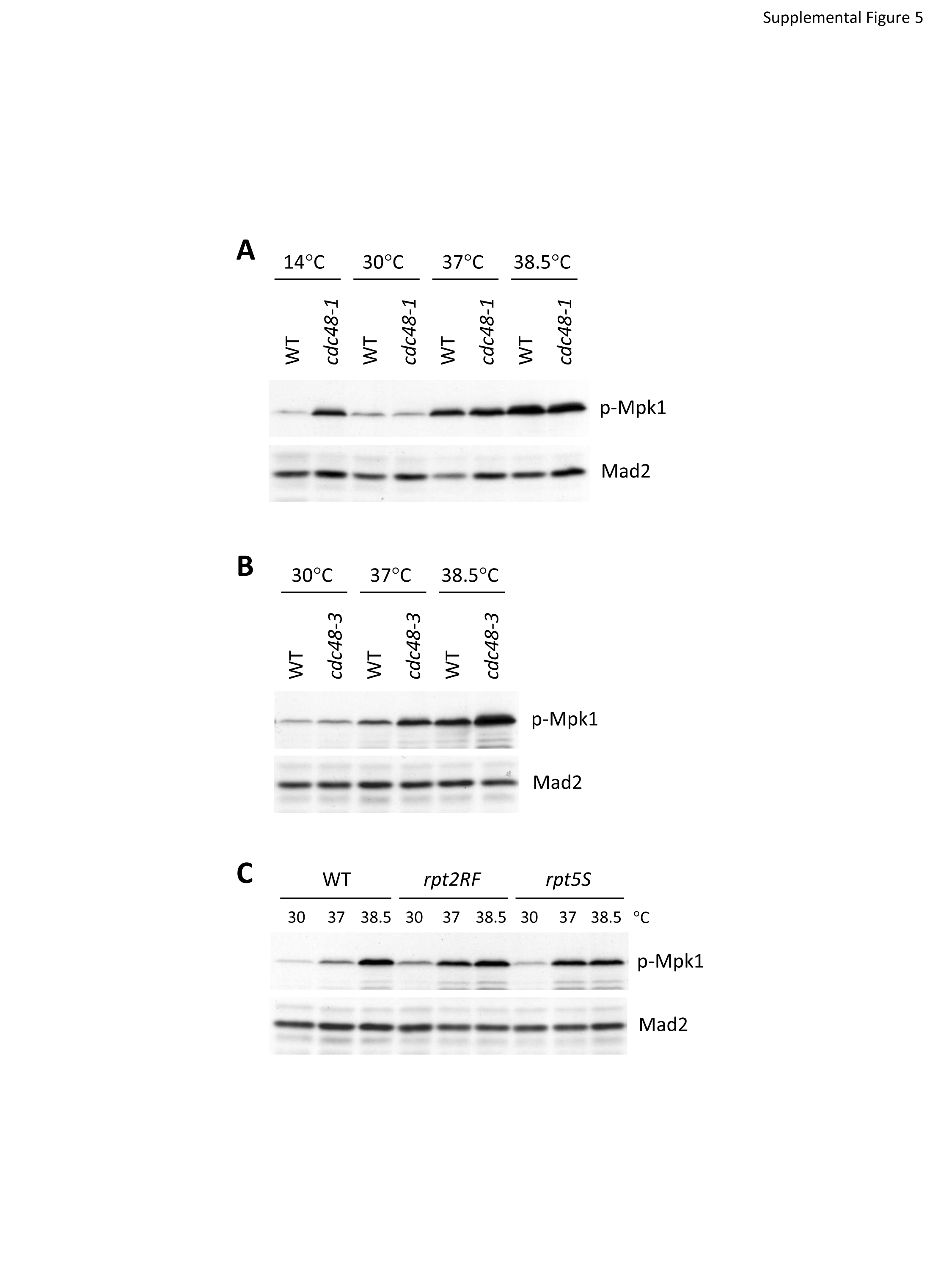

Supplement: Figure S5 — Phosphorylation of Mpk1 is enhanced in cdc48 mutants, but not in proteasome mutants. Cells of indicated genotypes were first grown at 25°C and then shifted to 14°C for 2 days or other indicated temperatures for 3 hr. Cell lysates were prepared for Western blots with anti-phospho-Mpk1 and anti-Mad2 antibodies. Mad2 serves as a loading control. (TIF) [file pone.0018988.s005.tif]
